# Estimating the apparent transverse relaxation time (R2^*^) from images with different contrasts (ESTATICS) reduces motion artifacts

**DOI:** 10.3389/fnins.2014.00278

**Published:** 2014-09-10

**Authors:** Nikolaus Weiskopf, Martina F. Callaghan, Oliver Josephs, Antoine Lutti, Siawoosh Mohammadi

**Affiliations:** ^1^Wellcome Trust Centre for Neuroimaging, UCL Institute of Neurology, University College LondonLondon, UK; ^2^Birkbeck-UCL Centre for NeuroImagingLondon, UK; ^3^Laboratoire de Recherche en Neuroimagerie, Department of Clinical Neurosciences, Centre Hospitalier Universitaire VaudoisLausanne, Switzerland

**Keywords:** T2^*^, transverse relaxation, apparent transverse relaxation, robust fit, multiple contrasts, motion, artifacts, quantitative imaging

## Abstract

Relaxation rates provide important information about tissue microstructure. Multi-parameter mapping (MPM) estimates multiple relaxation parameters from multi-echo FLASH acquisitions with different basic contrasts, i.e., proton density (PD), T1 or magnetization transfer (MT) weighting. Motion can particularly affect maps of the apparent transverse relaxation rate R2^*^, which are derived from the signal of PD-weighted images acquired at different echo times. To address the motion artifacts, we introduce ESTATICS, which robustly estimates R2^*^ from images even when acquired with different basic contrasts. ESTATICS extends the fitted signal model to account for inherent contrast differences in the PDw, T1w and MTw images. The fit was implemented as a conventional ordinary least squares optimization and as a robust fit with a small or large confidence interval. These three different implementations of ESTATICS were tested on data affected by severe motion artifacts and data with no prominent motion artifacts as determined by visual assessment or fast optical motion tracking. ESTATICS improved the quality of the R2^*^ maps and reduced the coefficient of variation for both types of data—with average reductions of 30% when severe motion artifacts were present. ESTATICS can be applied to any protocol comprised of multiple 2D/3D multi-echo FLASH acquisitions as used in the general research and clinical setting.

## Introduction

Quantitative magnetic resonance imaging (qMRI) provides standardized information about the tissue microstructure that can be compared across time points and imaging sites (e.g., Tofts, 2003; Schmierer et al., 2004; Dick et al., 2012; Weiskopf et al., 2013). Quantitative multi-parameter mapping (MPM) is a fast method for mapping the longitudinal relaxation rate R1, effective proton density PD^*^ (Lin et al., 1997; Weiskopf et al., 2013), magnetization transfer saturation (MT) and apparent transverse relaxation rate R2^*^ across the entire brain. These parameters are estimated from three differently weighted datasets acquired with a 3D fast low angle shot (FLASH) sequence using established physical models (Helms et al., 2008a,b; Weiskopf et al., 2013). The method was shown to provide important information about tissue microstructure, including tissue myelination in health and disease (Draganski et al., 2011; Dick et al., 2012; Freund et al., 2013; Sereno et al., 2013; Lutti et al., 2014) or iron concentration (Ordidge et al., 1994; Langkammer et al., 2010; Draganski et al., 2011; Callaghan et al., 2014a), which is based on the measurement of R2^*^.

The R2^*^ maps are particularly prone to motion artifacts, since their estimation requires images acquired at long echo times that are more affected by motions [TE ~ > 10 ms; (Versluis et al., 2010; Magerkurth et al., 2011)]. The other quantitative maps (R1, MT, PD^*^) generated from the MPM protocol are significantly less affected by motion, since they are estimated from averages across multiple echo times (including short echo times, Weiskopf et al., 2013).

Different methods were developed to address motion-related artifacts in high-resolution anatomical imaging. Standard retrospective rigid body motion correction cannot account for intra-scan motion artifacts and thus is less useful for long anatomical imaging (Friston et al., 1995; Kochunov et al., 2006). Most approaches correcting for intra-scan motion require modified pulse sequences, bespoke image reconstruction methods (Pipe, 1999; Versluis et al., 2010) or access to raw k-space data (Bydder et al., 2003; Lin et al., 2007; Magerkurth et al., 2011; Nöth et al., 2014). Prospective motion correction based on fast optical tracking provides a high quality flexible correction approach but it requires specialized hardware and software (Zaitsev et al., 2006; Maclaren et al., 2012). Frequently, the correction methods are not readily available on clinical MRI scanners. Also, the majority were developed for 2D imaging rather than 3D imaging that is usually used for high-resolution anatomical imaging.

We propose a novel method for correcting motion artifacts and increasing signal-to-noise ratio (SNR) in the R2^*^ maps estimated from 3D MPM modulus image data or similar protocols with multiple acquisitions: ESTimating the Apparent Transverse relaxation time (R2^*^) from Images with different ContrastS (ESTATICS). The MPM method estimates R2^*^ from the echoes of the PD-weighted FLASH acquisition only. ESTATICS extends the method to using data from all three differently weighted acquisitions by accounting for the varying contrasts. We also complement the standard ordinary least squares (OLS) log-linear fit with a robust fitting procedure, which detects and down weights echoes affected by motion artifacts. We demonstrate the validity and effectiveness of ESTATICS in data with no prominent motion artifacts and data affected by severe motion artifacts. In addition to testing on typical data acquired in a neuroimaging center, fast optical motion tracking and prospective motion correction is employed to define a motion artifact-free gold standard.

## Methods

### Estatics

The MPM protocol yields three multi-echo FLASH datasets with PD-, T1- and MT-weighting and between 6 and 8 echoes per dataset at different echo times TE: *I_PD_*(*TE*), *I_T1_*(*TE*), and *I_MT_*(*TE*). Assuming mono-exponential signal decay with TE, the signal equation is (here for the PD-weighted scan):

(1) $$I_{PD}\left(TE\right)\;=\;I_{PD}\left(0\right)e^{-R_{2}^{*}TE}$$

with *I_PD_*(0) being the signal at TE = 0 determined by the net magnetization and sensitivity of the MR system. Note that we also assume that R^*^_2_ is constant across the different FLASH acquisitions, an assumption that is tested in this study. The standard MPM method estimates ${\hat{I}}_{PD}(0)$ and ${\hat{R}}_{2}^{*}$ by a log-linear fit of the PD-weighted data, i.e., minimizing the error ε of:

(2) $$\varepsilon \;=\;\sum_{n\;=\;1}^{n_{max}}{\left[ln\left(I_{PD}\left(TE\left(n\right)\right)\right)+{\hat{R}}_{2}^{*}TE\left(n\right)-\text{ln}\left({\hat{I}}_{PD}\left(0\right)\right)\right]}^{2}$$

with n being the echo number and *n_max_* the maximal number of echoes.

For the ESTATICS approach, we generalize this optimization problem to three or more contrasts by simultaneously optimizing across all contrasts minimizing the error ε now defined as:

(3) $$\varepsilon \;=\;\sum_{k=1}^{k_{max}}\;\sum_{n=1}^{n_{max}(k)}{\left[ln\left(I_{k}\left(TE\left(n\right)\right)\right)+{\hat{R}}_{2}^{*}TE\left(n\right)-\text{ln}\left(\;{\hat{I}}_{k}\left(0\right)\right)\right]}^{2}$$

with k being the contrast number (e.g., for PD-weighting or T1-weighting), *k_max_* the maximal number of contrasts and *n_max_*(*k*) the maximal number of echoes for contrast *k*.

Note that the different contrast weighting is accounted for by different contrast specific ${\hat{I}}_{k}(0)$ and that R2^*^ is assumed to be independent of the contrast weighting, i.e., resulting in only a single ${\hat{R}}_{2}^{*}$ estimate.

The generalized optimization problem (Equation 3) was solved with established OLS (e.g., Aster and Thurber, 2012) and robust fitting approaches. The robust fitting approach, which is routinely used in other quantitative imaging modalities (e.g., Mohammadi et al., 2013a), is further decribed in the Data Analysis Section.

### Data acquisition

Two studies assessed the performance of the different R2^*^ estimation approaches. The first study was based on a group of volunteers representing the typical population at a cognitive neuroimaging center. The second study assessed the performance in a single volunteer with precise tracking of motion trajectories using an optical motion tracking system.

#### Group study

The data of 20 healthy volunteers (10 female, mean age 22.6 years, std. dev. 2.9 years) acquired as part of studies conducted within the cognitive neuroimaging program at the Wellcome Trust Centre for Neuroimaging were analyzed. The studies were approved by the local ethics committee and informed written consent was obtained prior to scanning. Although volunteers were instructed to minimize head motion as part of the routine pre-scan briefing, severe head motion artifacts were visible in some volunteers' data. Two groups of 10 datasets were visually selected so that one group was corrupted by severe motion artifacts (*large-motion group*) while the other was free of prominent motion artifacts (*small-motion group*). The two groups were also matched for age and gender.

Whole-brain quantitative MPM data (Weiskopf et al., 2013) were acquired on a 3T whole body MRI scanner (Magnetom TIM Trio, Siemens Healthcare, Erlangen, Germany) equipped with a standard 32 channel head coil for receive and radiofrequency (RF) body coil for transmission. The details of the data acquisition are reported in Weiskopf et al. (2013) and are briefly summarized here for convenience. The three different multi-echo FLASH scans were acquired with predominant T1-, PD-, and MT-weighting by specific choice of the repetition time (TR) and the flip angle α: TR/α = 18.7 ms/20° for the T1w scan and 23.7 ms/6° for the PDw and the MTw scans. MT-weighting was achieved by applying an off-resonance Gaussian-shaped RF pulse (4 ms duration, 220° nominal flip angle, 2 kHz frequency offset from water resonance) prior to the excitation. Multiple gradient echoes were acquired with alternating readout polarity at six equidistant echo times (TE) between 2.2 and 14.7 ms for the T1w and MTw acquisitions and at 8 equidistant TE between 2.2 and 19.7 ms for the PDw acquisition. Thus, following the notation in the Theory section, the number of contrasts was *k_max_* = 3, and the corresponding number of echoes were *n_max_*(T1) = *n_max_*(MT) = 6, *n_max_*(PD) = 8.

Other acquisition parameters were: 1 mm isotropic resolution, 176 sagittal partitions, field of view (FOV) = 256 mm × 240 mm, parallel imaging using GRAPPA factor 2 in phase-encoding (PE) direction, 6/8 partial Fourier in partition direction, non-selective RF excitation, readout bandwidth BW = 425 Hz/pixel, RF spoiling phase increment = 50°, total acquisition time for the FLASH sequences ~ 19 min. Additional reference data for correction of RF transmit field inhomogeneities (Lutti et al., 2012) were acquired. The total scanning time of the MPM protocol was approximately 25 min.

#### High-resolution R2^*^ mapping with optical motion tracking

To characterize the relation between particular motion trajectories and the artifact level in R2^*^ maps, a single healthy volunteer (male, age 45 years) was scanned on the same MRI scanner setup but with an additional optical motion tracking system (Kineticor, HI; Maclaren et al., 2012). The study was approved by the local ethics committee and informed written consent was obtained prior to scanning.

The optical motion tracking system provided and recorded information about the head position with high precision on the order of tens of microns at 85 Hz frame rate. Using special software libraries (Zaitsev et al., 2006; Herbst et al., 2012, 2014), the information could also be used to dynamically update the imaging volume such that it followed the head, i.e., for prospective motion correction (PMC).

The MPM data acquisition was similar to the protocol used in the group study but achieved an isotropic resolution of 800 μm instead of 1 mm. The three different multi-echo FLASH scans were acquired with predominant T1-, PD-, and MT-weighting by specific choice of the repetition time (TR) and the flip angle α: TR/α = 27 ms/21° for the T1w scan and 27 ms/6° for the PDw and the MTw scans. MT-weighting was achieved by applying an off-resonance Gaussian-shaped RF pulse as described above. Multiple gradient echoes were acquired with alternating readout polarity at six equidistant echo times (TE) between 2.34 and 13.84 ms for the MTw acquisitions and at 8 equidistant echo times for the T1w and PDw acquisitions with TE between 2.34 and 18.44 ms.

The other acquisition parameters were: 208 sagittal partitions, field of view (FOV) = 256 × 224 mm, parallel imaging using GRAPPA factor 2 in phase-encoding (PE) direction, 6/8 partial Fourier in partition direction, non-selective RF excitation, readout bandwidth BW = 488 Hz/pixel, RF spoiling phase increment = 137°, total acquisition time for the FLASH sequences ~ 32 min. Additional reference data for correction of RF transmit field inhomogeneities (Lutti et al., 2012) were acquired. The total scanning time of the MPM protocol was approximately 40 min. The PDw acquisition was repeated with the PMC system turned on (i.e., tracking and correcting for motion) and off (i.e., tracking but not correcting for motion). The volunteer was then allowed to move freely during an additional acquisition with the PMC system off.

### Data analysis

#### Group study

Data were analyzed using tools implemented in Matlab (The Mathworks) including SPM8 (Friston et al., 2007) and the VBQ toolbox for analyzing MPM data (Draganski et al., 2011; Weiskopf et al., 2013), which was extended to include the ESTATICS approach. The different acquisitions were coregistered for each participant. T1-weighted images were segmented into different tissue classes including gray and white matter using the unified segmentation approach (Ashburner and Friston, 2005). The non-linear transform from single subject space to MNI space was estimated also using unified segmentation. For quantitative analysis, a frontal region-of-interest (ROI) was defined in MNI space and transformed into the individual subject space using the inverse non-linear transform. The ROI encompassed a cube with face length 10 mm in each hemisphere. Only voxels with a white matter probability higher than 95% within the ROI were included in the analysis.

The R2^*^ maps were estimated in six different ways for assessment of the ESTATICS method. Three estimations were based on an ordinary least squares (*OLS*) fit of the single PDw, T1w or MTw scan. The other three estimations used the three weighted scans in combination and were based on an OLS fit and two different types of robust fits.

As a reference, OLS fits were performed separately for the multi-echo data of the PDw (=standard approach, denoted R^*^_2_(PDw)), T1w (denoted R^*^_2_(T1w)) and MTw (denoted R^*^_2_(MTw)) scans. For ESTATICS, the multi-echo data combined across the three different weightings were modeled using OLS (denoted R^*^_2_(OLS)) and robust fitting. The robust-fitting approach down-weighted outliers and corrected the distortion of the residual error distribution from using the logarithm of the signal in Equation (1). The confidence interval that determined whether data points were within the noise variation or should be treated as outliers was estimated from the distribution of the residuals of the model fit using the standard procedure (for details see e.g., Meer et al., 1991; Mangin et al., 2002).

We used two different approaches to calculate the weight functions for the outlier rejection: the first approach used a voxel-wise varying weighting function (similar to Holland and Welsch, 1977), whereas the second approach factorized the weighting function into a voxel-wise, a line-wise, and a plane-wise varying component (similar to the procedures described in Zwiers, 2010; Mohammadi et al., 2013a,b). The first method resulted in a normal SNR but was less robust against outliers, whereas the second method was more robust against outliers but resulted in a lower SNR. We denote the former method *high SNR robust fitting* (R^*^_2_(Robust Fitting High SNR)) and the latter *robust fitting* (denoted R^*^_2_(Robust Fitting)) in the following.

For quantitative assessment, the mean and standard deviation (sd) across voxels, and the coefficient of variation (CoV = sd/mean) within the frontal white matter ROI were determined for each volunteer and estimation method. The validity of this particular measure of CoV depends on negligible physiological variations within the ROI (Helms et al., 2009; Weiskopf et al., 2013). Therefore, the ROI was limited to a small cube containing white matter only, providing a conservative estimate of the noise level.

Statistical analysis was performed to test for differences in CoV depending on the fitting method. The analysis was conducted separately for the small-motion and large-motion group of volunteers, since the statistical distributions were expected to be considerably different. Since the data affected by motion was not Gaussian distributed, a non-parametric two-tailed Wilcoxon signed rank test was used to test for pair-wise significant differences between the OLS fit based on the PDw images and the other five different estimation methods. In addition, the same test was used to test for pair-wise differences between ESTATICS based on the OLS and the two robust fitting methods. A conservative statistical threshold of *p* < 0.05/8 = 0.0063 was used to correct for multiple comparisons within each group of volunteers (8 tests in total per group).

To test for bias induced by motion artifacts, the percent deviations of R2^*^ estimates between the large-motion group and the small-motion group were estimated for each method separately. A two-tailed Wilcoxon signed rank test with a liberal statistical threshold of *p* < 0.05 determined significance, to achieve a high sensitivity to any bias.

To assess a potential bias of the alternative R2^*^ estimation approaches, the percent difference between each method-specific R2^*^ estimate and the current standard approach (R^*^_2_(PDw)) was determined.

#### High-resolution R2^*^ mapping with optical motion tracking

R2^*^ maps of the high-resolution data were estimated with the same methods as in the group study. The quality of the maps was determined by careful visual inspection. To assess the relation between motion and artifacts, a summary scalar metric capturing the rate of motion per TR was estimated from the motion tracking data by numerical differentiation and square root sum of squares combination of the rotation (pitch, roll, yaw) and translation (x, y, z) measures.

## Results

**Figure 1:**
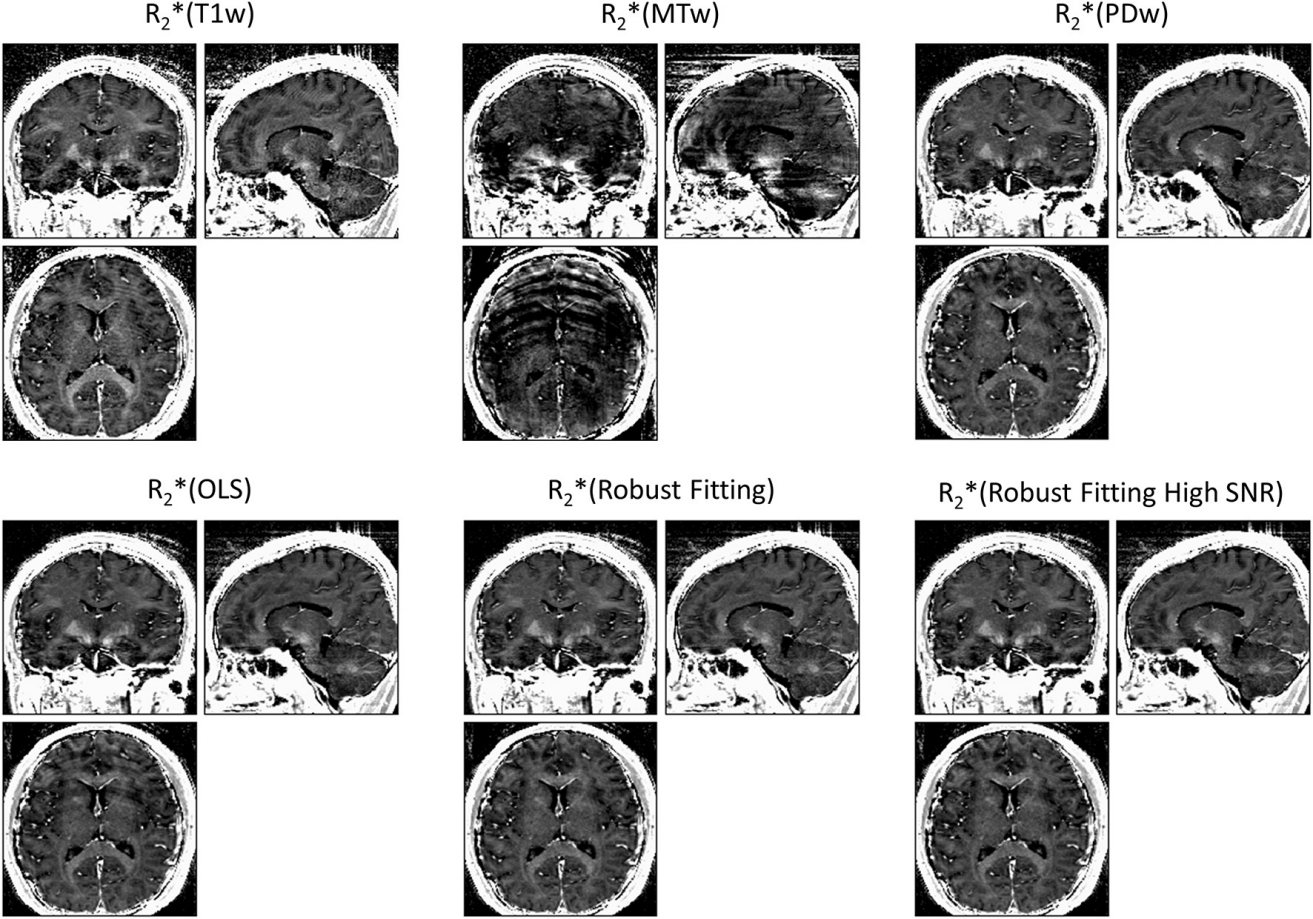
Correction of motion artifacts in R2^*^ maps estimated from multi-echo FLASH data. The top row shows maps generated from multiple echoes of a single primary contrast weighting, i.e., R2^*^(T1w), R2^*^(MTw) or R2^*^(PDw) (= standard method) maps. The bottom row shows maps estimated with the ESTATICS approach, i.e., R2^*^(OLS), R2^*^(Robust Fitting) and R2^*^(Robust Fitting High SNR). ESTATICS significantly reduced the motion artifacts seen in the R2^*^(MTw) map. All maps are windowed from 5 to 50 s^−1^.

**Figure 2:**
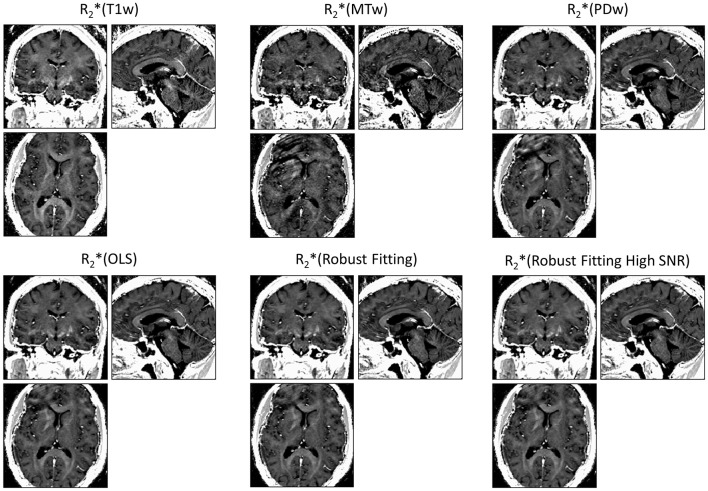
Correction of motion artifacts in R2^*^ maps estimated from multi-echo FLASH. ESTATICS significantly reduced the motion artifacts seen in R2^*^(MTw) and R2^*^(PDw) maps. Since two contrast weightings were affected by motion, the motion correction was less effective than when a single contrast weighting was affected (see Figure 1), e.g., some artifacts remained in the frontal cortex. See Figure 1 for further details. All maps are windowed from 5 to 50 s^−1^.

**Figure 3:**
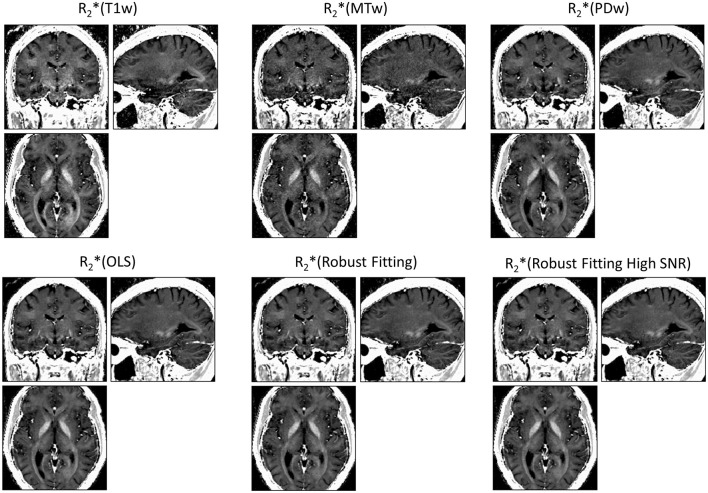
R2^*^ maps estimated from multi-echo FLASH data unaffected by motion. ESTATICS improved the SNR and delineation of contrast edges even when there were no significant motion artifacts apparent. See Figure 1 for further details. All maps are windowed from 5 to 50 s^−1^.

ESTATICS significantly reduced motion artifacts in the R2^*^ maps as can be seen in Figure 1 for a volunteer from the large-motion group. Severe ringing artifacts occurred in the R^*^_2_(MTw) map (top center, Figure 1) but were not present in the robust fitting ESTATICS estimates R^*^_2_(Robust Fitting) and R^*^_2_(Robust Fitting High SNR); (bottom center and right) and were also reduced in the OLS ESTATICS estimate R^*^_2_(OLS) (bottom left). If several contrasts were affected by motion, ESTATICS did not achieve a complete correction of artifacts as can be seen in a case where the MTw and PDw images were affected and some artifacts in the frontal cortex remained (Figure 2). The R2^*^ map quality was improved even in the small-motion group unaffected by prominent motion artifacts as can be seen in Figure 3. In particular the SNR and definition of contrast edges were improved.

**Figure 4:**
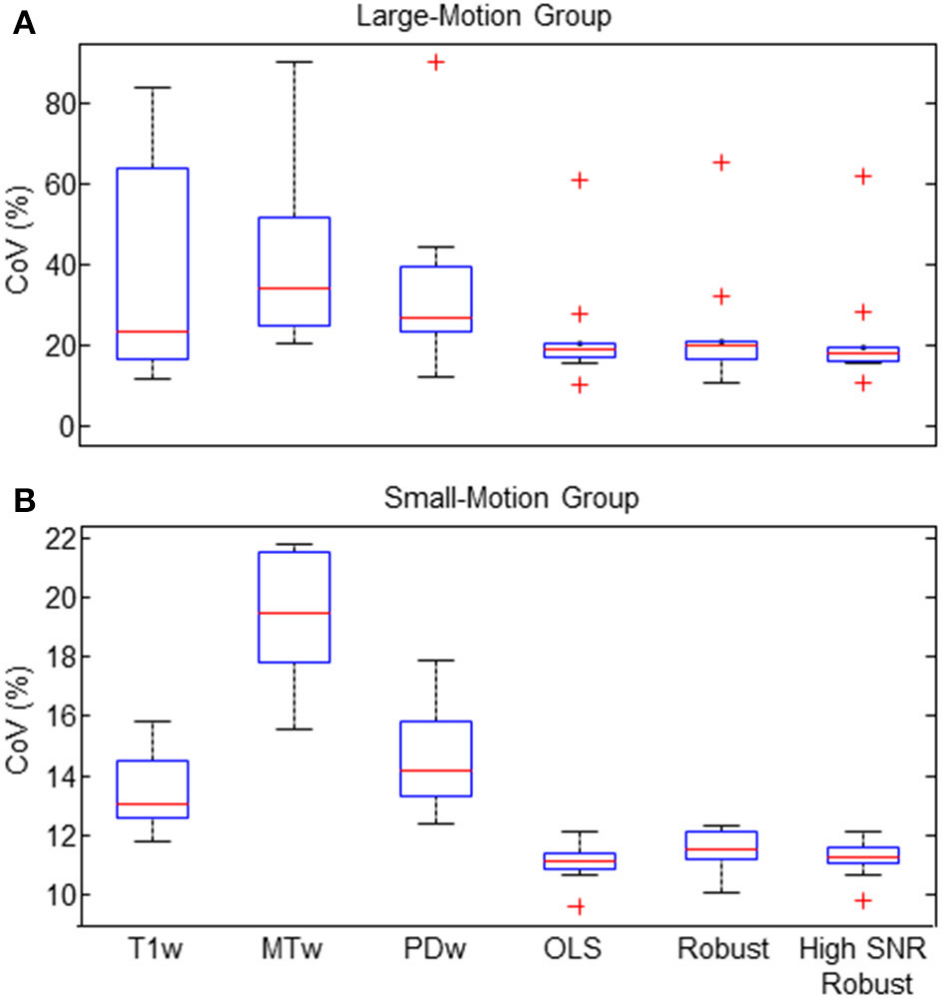
Coefficient of varation (CoV) of R2^*^ was reduced by ESTATICS (as measured in the frontal white matter ROI). **(A)** In the large-motion group the CoV was considerably reduced using ESTATICS. Also the consistency between volunteers was increased as can be seen by reduced inter-subject variation. **(B)** In the small-motion group the CoV was also reduced using ESTATICS but the absolute effect was smaller. Box plots provide descriptive statistics for CoV across the group with blue box = 25/75% percentile, red line = median, black whisker = most extreme data value excluding outliers, red cross = outlier (probability < 0.01 under assumption of normally distributed data). Note the different scale of the y-axis for the data of small-motion and large-motion groups. See Figure 1 for explanation of abbreviations.

The CoV in the frontal white matter ROI was significantly decreased for all three different ESTATICS methods compared to the standard approach (R^*^_2_(PDw)) for both groups of volunteers (Figures 4A,B; all *p* < 0.0059; all *W* ≤ 2). For the large-motion group, there was no significant difference between R^*^_2_(OLS), R^*^_2_(Robust Fitting) and R^*^_2_(Robust Fitting High SNR) when accounting for multiple comparisons (Figure 4A; all *p* > 0.011, all *W* ≥ 2). For the small-motion group, the CoV was increased for the R^*^_2_(Robust Fitting) method compared to the R^*^_2_(OLS) and R^*^_2_(Robust Fitting High SNR) methods (Figure 4B; both *p* < 0.0039; both *W* = 0).

**Figure 5:**
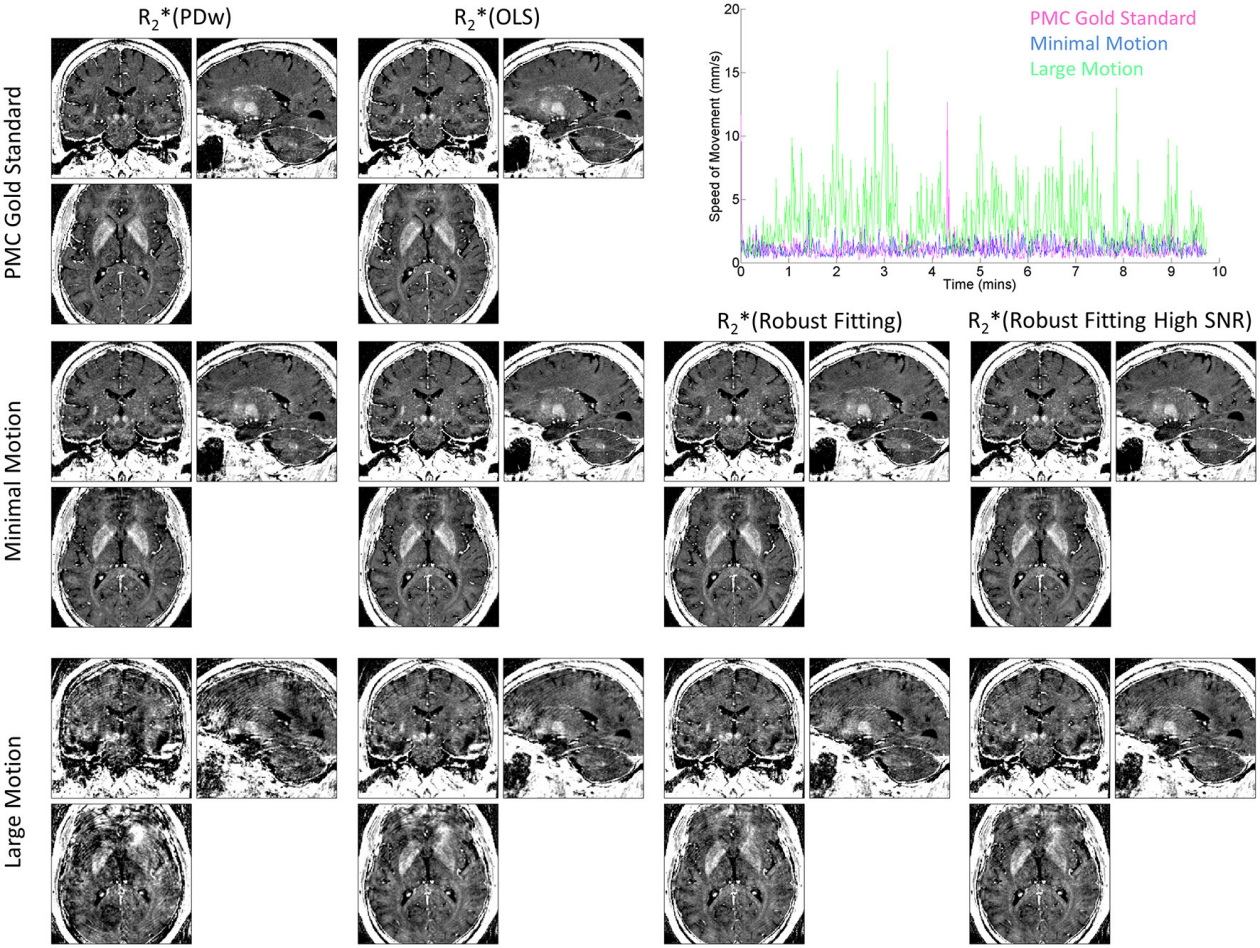
R2^*^ maps at 800 μm isotropic resolution and corresponding motion trajectories in a single volunteer. High-speed optical motion tracking determined head motion and allowed for prospective motion correction (PMC). R2^*^ maps were estimated with different methods. First column shows R2^*^(PDw), the last three columns show the different ESTATICS implementations. Rows show data with increasing levels of motion artifacts. First row shows data acquired with PMC and serves as the no motion artifact gold standard. Second row shows data acquired with minimal head motion without PMC. Third row shows data affected by motion during the PDw image acquisition without PMC. Top right panel shows speed of motion traces for the PDw image acquisitions for the three conditions.

The improvement of R2^*^ maps by ESTATICS was also clearly visible in the high-resolution dataset (Figure 5, left column vs. three right columns in the bottom row). In particular, the frontal brain regions showed considerably reduced artifact levels compared to the standard approach. Ringing was reduced and homogeneity of the R2^*^ maps was improved. The motion tracking (Figure 5, top right panel) confirmed extensive head motion in the large motion case and minimal movement in the other cases.

Motion induced bias was assessed by comparing the R2^*^ estimates in the large-motion group with the small-motion group (in the frontal ROI; note—no figure shown). There was no significant difference in the amount of motion induced bias for the different methods when compared to the standard approach (all *p* > 0.12; all *W* ≥ 11.5). However, the R2^*^ (Robust Fitting) method showed a lower deviation when directly compared to the R^*^_2_(OLS) and R^*^_2_(Robust Fitting high SNR) methods (both *p* < 0.039; both *W* ≤ 7.5), indicating a higher robustness and accuracy of the robust fitting.

**Figure 6:**
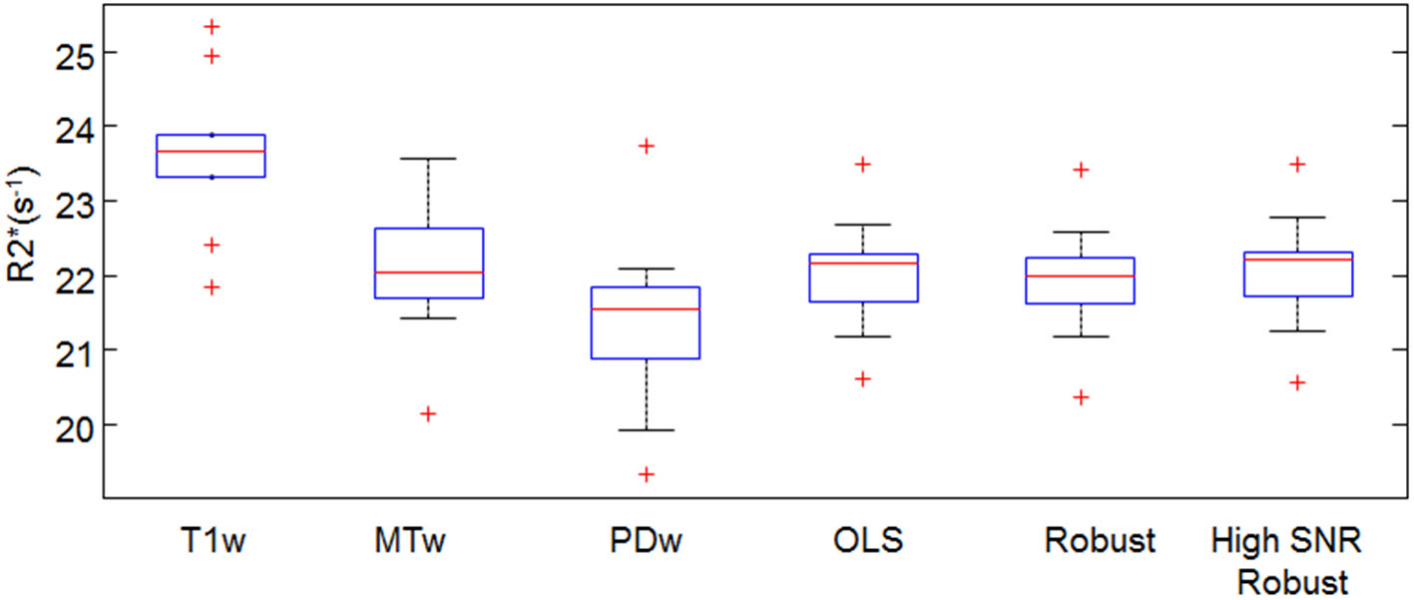
Mean R2^*^ in the frontal white ROI estimated by different fitting approaches. For definition of abbreviations and box plots see Figures 1,4.

The absolute R2^*^ estimates in the frontal ROI depended on the fitting method (Figure 6). For example in the small-motion group, the R2^*^(T1w) estimates were 9.5 ± 9.1% (median±inter quartile range) higher than R^*^_2_(PDw), and the R^*^_2_(MTw) estimates were 2.3 ± 10.5% higher than R^*^_2_(PDw). When using ESTATICS, the R2^*^ was increased by 3.0 ± 5.2%, 2.6 ± 3.8% and 3.1 ± 5.1%, for R^*^_2_(OLS), R^*^_2_(Robust Fitting) and R^*^_2_(Robust Fitting High SNR) respectively.

## Discussion

Motion artifacts in R2^*^ maps were significantly reduced and the SNR was significantly increased by ESTATICS—a novel approach to fitting the multi-echo data from FLASH acquisitions with different contrast weighting, as e.g., acquired for quantitative multi-parameter mapping (MPM; Weiskopf et al., 2013). The coefficient of variation of R2^*^ estimates was reduced by ca. 30% in data affected by motion (Figure 4). The homogeneity and appearance of R2^*^ maps were clearly improved (Figures 1–3,5).

Motion artifacts in long echo time acquisitions are a limiting factor for R2^*^ mapping, particularly in non-compliant volunteers or patients (Versluis et al., 2010; Nöth et al., 2014). For example, in Parkinson's disease R2^*^ maps can be difficult to measure due to involuntary head motion but they are an important tissue probe due to known iron concentration changes.

Several methods were developed to avoid and correct for motion artifacts in high resolution anatomical imaging. Retrospective rigid body motion correction (Friston et al., 1995; Kochunov et al., 2006) can be used for correcting inter-scan motion but does not address intra-scan motion, which is particularly problematic for the long 3D acquisitions used for anatomical imaging. The majority of methods correcting for intra-scan motion require modified pulse sequences, advanced image reconstruction methods (Pipe, 1999; Versluis et al., 2010), access to raw k-space data (Bydder et al., 2003; Lin et al., 2007; Magerkurth et al., 2011; Nöth et al., 2014) or even specialized hardware (Maclaren et al., 2012). Handling of raw k-space is difficult in large scale research and clinical scanning. Most intra-scan motion correction methods were not designed for and may not be applicable to 3D acquisitions that are frequently used in high-resolution anatomical scanning due to their higher signal-to-noise ratio and high through-slice resolution (e.g., Pipe, 1999; Magerkurth et al., 2011; Nöth et al., 2014).

Prospective motion correction based on fast optical tracking has great promise for flexible and effective motion correction but depends on additional hardware and stable tracking marker fixation (Maclaren et al., 2012). MR pulse sequences acquiring rotating blades in k-space, such as PROPELLER (Pipe, 1999), are usually only implemented for 2D multi-slice imaging, limiting the correction of 3D motion and also their application to 3D MRI acquisitions. The PROPELLER approach (Pipe, 1999) and also navigator based correction (Versluis et al., 2010) further require extra scans, modified pulses sequences and advanced image reconstruction. Alternative autofocusing techniques require access to raw k-space data and bespoke reconstruction methods (Lin et al., 2007).

Motion correction methods were also tailored to R2^*^ mapping using 2D FLASH acquisitions (Magerkurth et al., 2011; Nöth et al., 2014). They are based on repeated acquisitions of different central k-space parts and optimization of data consistency across scans with respect to a mono-exponential signal decay across echo time. Considerable improvements in scan quality were demonstrated for healthy volunteers and patients (Magerkurth et al., 2011; Nöth et al., 2014). However, the methods were only developed for 2D multi-slice acquisitions and cannot combine data acquired with different contrasts such as MPM datasets. They also rely on access to k-space raw data.

Compared to the previous correction methods, ESTATICS is a genuine post-processing method and only requires modulus image data as, e.g., acquired by multi-echo FLASH sequences and usually available in the clinical environment. It is compatible with both 2D multi-slice and 3D data acquisition. In combination with the established MPM protocol it offers a comprehensive tool for robust quantitative imaging and assessment of tissue microstructure (Draganski et al., 2011; Weiskopf et al., 2011, 2013; Dick et al., 2012; Freund et al., 2013; Sereno et al., 2013; Callaghan et al., 2014a,b; Lutti et al., 2014). We would like to note that motion artifacts usually affect the R2^*^ maps more severely than the other MPM maps that are estimated from averages of multiple images at different echo times (Weiskopf et al., 2013).

The ESTATICS approach improves SNR and contrast definition in the R2^*^ maps relative to the standard single contrast R2^*^ estimation approach since it utilizes data from all contrasts. The robust fitting methods may reduce the SNR in the R2^*^ maps compared to OLS fitting, since it intrinsically reduces the degrees of freedom in the dataset. Especially data with high SNR and minimal artifact level may benefit from using OLS fitting. To alleviate this issue, the confidence interval of the robust fitting algorithm can be tuned to optimize for SNR and ignore low-amplitude outliers—as done in this study. However, in our study the SNR difference between OLS and robust fitting was almost negligible compared to the general improvement due to ESTATICS as can be seen in Figure 4B.

ESTATICS is based on the assumption that R2^*^ is constant across the different FLASH acquisitions. Strictly, this is not correct when the FLASH data are acquired with different T1 or MT weightings, since different tissue compartments contribute differently to the FLASH signal depending on the weighting. For example, R2^*^ estimates from T1-weighted images were increased by approximately 9.5%, since presumably the relative contribution from myelin water was increased, which has a higher R2^*^ than other tissue. Tests in a doped water phantom (not shown) resulted in maximal deviations of 2.5% in R2^*^ using the different weightings. Although the phantom tests do not fully exclude the possibility of technical artifacts introducing bias, they indicate that they are not the major source of the bias. ESTATICS using the entire dataset showed a much smaller difference of ca. 3% from the standard R2^*^ estimates based on the PD-weighted images only. Although this difference is small, the possibility of bias should be considered when interpreting or comparing results across studies. We are developing biophysical models, such as the linear relaxometry model (Callaghan et al., 2014b), which may help in reducing this bias in the future since they can in principle account for the interaction between the free induction decay and contrast weighting in FLASH images.

Our implementation of ESTATICS assumes mono-exponential free induction decay in line with previous correction approaches (Magerkurth et al., 2011; Nöth et al., 2014). This assumption will be violated in brain areas suffering from significant susceptibility artifacts (Posse et al., 2003; Neeb et al., 2006) or partial volume effects. The rather high ≤1 mm isotropic resolution used in our study reduced the impact of susceptibility artifacts and partial volume effects on the R2^*^ maps but imaging protocols with lower resolution may be more affected. It is also known that non mono-exponential decays occur due to intrinsic properties of myelination (Wharton and Bowtell, 2012). However, these effects are expected to be rather small compared to the aforementioned susceptibility artifacts.

If the SNR in the multi-echo data is low, the fitting of the log-linear modulus signal is suboptimal and will lead to bias due to the non Gaussian noise distribution. We did not observe this problem in our data but lower SNR acquisitions, for example, at higher resolution or longer echo times, may be affected.

In principle, the multi-echo data may be combined using various alternative strategies. We chose established OLS and robust fitting methods for their well known characteristics and description. However, further improvements may be possible, e.g., by tailoring the data combination approach for specific types of motion. As an example for such an optimization, we implemented a high SNR robust fitting variant. We would like to note that simply averaging the R2^*^ maps from the different basic contrasts (i.e., averaging R^*^_2_(MTw), R^*^_2_(PDw) and R^*^_2_(T1w)) resulted in a higher bias in the R2^*^ estimates within the frontal white matter ROI than robust or OLS fitting (data not shown).

Assessment of motion correction methods relies on test motion trajectories that are realistic for the scanned target groups of patients and volunteers and ideally are precisely known. So far, knowledge about typical head motion in patients and volunteers is limited. To capture realistic motion in healthy volunteers, we manually selected large- and small-motion groups from studies conducted within the cognitive neuroimaging program at our center. In addition, we used a fast and precise optical tracking system in one volunteer, to allow for the exact characterization of the head motion (Figure 5). However, we did not test our method in patients as some previous studies (Versluis et al., 2010; Magerkurth et al., 2011; Nöth et al., 2014). The typical motion trajectories may differ between volunteers and patients but also between different patient populations, warranting further studies for their characterization. This characterization will be facilitated by the advent of fast and non interfering optical tracking systems.

ESTATICS efficiently addresses the artifacts caused by head motion in R2^*^ maps derived from multiple FLASH acquisitions. Since it is a post-processing method that does not require tailored pulse sequences and is applied to standard modulus image data, it can be readily applied to 2D and 3D FLASH data widely accessible in research and clinics. In combination with quantitative multi-parameter mapping it enables robust, fast, whole brain, high resolution mapping of image parameters and tissue microstructure.

### Conflict of interest statement

The Wellcome Trust Centre for Neuroimaging has an institutional research agreement with Siemens and receives support from Siemens. The reviewer, Dr Deichmann, declares that despite having collaborated with the authors on previous occasions the review process was handled objectively.
